# Quercetin can reduce viral RNA level of O’nyong-nyong virus and resulting innate immune cytokine responses in cultured human synovial fibroblasts

**DOI:** 10.1038/s41598-021-85840-z

**Published:** 2021-03-18

**Authors:** Axelle Septembre-Malaterre, Yosra Bedoui, Claude Giry, Philippe Gasque, Pascale Guiraud, Jimmy Sélambarom

**Affiliations:** 1 Université de La Réunion, Unité de recherche Etudes Pharmaco-Immunologie (EPI), CHU La Réunion site Félix Guyon, Allée des Topazes, CS11021, 97400 Saint Denis de La, Réunion, France; 2 Laboratoire d’immunologie clinique et expérimentale de la zone de l’océan indien (LICE-OI, CHU La Réunion site Félix Guyon, Allée des Topazes, CS11021, 97400 Saint Denis de La, Réunion, France

**Keywords:** Biochemistry, Cell biology

## Abstract

O’nyong-nyong virus is an alphavirus closely related to chikungunya virus, causing arthralgia, rash and fever. Alphaviruses mainly target synovial fibroblasts and persists in the joints of patients, possibly leading to chronic arthritis. To date, no specific antiviral treatment is available for ONNV infection and induced-inflammation. Primary human synovial fibroblasts cells were used to assess infection by ONNV and the resulting cytokine responses. Phenolics (gallic acid, caffeic acid and chlorogenic acid, curcumin and quercetin) and a curcuminoids-rich extract from turmeric were tested for their antiviral and anti-inflammatory capacities. We showed that infection occurred in HSF cells and increased gene expression and protein secretion of two major proinflammatory CCL-2 and IL-1β markers. In ONNV-infected HSF cells (MOI 1), we found that non-cytotoxic concentrations of phenolics (10 µM) reduced the level of viral RNA (E1, E2, nsP1, nsP2) and downregulated CCL-2 and IL-1β expression and secretion. These results highlighted the high value of the flavonol quercetin to reduce viral RNA levels and inflammatory status induced by ONNV in HSF cells.

## Introduction

O'nyong-nyong virus (ONNV) is a mosquito-borne virus belonging to the genus *Alphavirus* in the *Togaviridae* family. This RNA virus, closely related antigenically, genetically and clinically to chikungunya virus (CHIKV), was first isolated in 1959 in Uganda during the first epidemic in East Africa which affected more than 2 million people^1^. ONNV is endemic in sub-Saharan Africa and its reemergence is reflected by several outbreaks (1984–1985, 1996–1997, 2003) in eastern and western Africa^2^. Vertebrate reservoir hosts of ONNV remain largely unknown though the virus is known to be transmitted by *Anopheles* mosquitoes, which also are vectors of malaria parasites^3^. The importation of ONNV to Europe by an infected traveler from Kenya in 2013 highlights the potential for worldwide introduction of ONNV by travelers^4^. As recently experienced with CHIKV, reemergence of ONNV with large outbreaks should be considered^5^.

ONNV causes symptoms similar to those caused by CHIKV including fever, rash and arthralgia^6–8^. Due to their antigenic and clinical similarities, the development of vaccines or drugs may serve in both cases^9^, as recently reported with a serogrouping-protection vaccine using mouse models^10^. In vivo studies in mouse model support the importance of innate responses in the initial control of ONNV infection, while the adaptive immune system mainly operates in the acute infection^5^. Chronic manifestations of ONNV infection are poorly understood^11^, there is evidence that alphavirus infection may cause persistent arthralgia and arthritis from residual virus in target cells and subsequent accumulation of inflammatory mediators^12,13^.

ONNV is classified as an arthritogenic alphavirus due to joint symptoms resulting from cartilage and bone damage^14^. In-depth investigations of alphavirus pathogenesis and immunity have established the critical role of synovial fibroblasts which are part of connective tissue (synovium) around human joints and are the primary target of alphavirus infection and its chronic manifestations^12^. Synovial fibroblasts are established as innate immune cells by recognizing invading pathogens via pattern recognition receptors (PRR)^15^ and are involved in alphavirus chronic manifestations and joint damage^16^. Furthermore, synovial fibroblasts are enrolled in cell signaling via interactions with other major immune cells including monocytes and macrophages^17^. These synovial-derived cells have therefore gained growing interest during the past decade to study in vitro alphavirus infection and management, with a recent attempt to clarify their role^18^.

To date, there are no available antiviral drugs or vaccines for the management of arthritogenic alphavirus infection^11^. Identification of antiviral effectors remains a continuous challenge^19,20^. The available treatment of symptomatic alphavirus disorders involves treatment with analgesics, antipyretics and non-steroidal anti-inflammatory drugs. More attention is being paid to the possible adverse effects of in-depth treatments. Methotrexate, the most common conventional disease modifying anti-rheumatic drug, is applied to relieve chronic CHIKV-induced arthritis, and without evidence for adverse immunological effects^21,22^. In contrast, the antimalarial chloroquine was used to control CHIKV replication but was found to increase chronic clinical manifestations^23^.

Natural immunomodulators have gained relevance for the treatment of non-immune and immune chronic inflammatory diseases^24^ and their development is supported by preclinical evaluation and clinical trials^25^. This new option includes naturally occurring phenolics^26^ also recognized as the most abundant antioxidants in human diet^27^ and established for various biological activities including anti-inflammatory, antiviral and anti-atherogenic effects^28–30^. The anti-inflammatory potential of phenolic compounds is exploited in folk medicine^31^ and mainly relies on their interference with the redox system and cytokine pathways^32^. From cumulative in vitro, in vivo and clinical data, the predominant innate immune responses against arthritogenic alphavirus infection involves a broad range of inflammatory mediators, as mainly evidenced from CHIKV but with a notable exception for ONNV^12,20^. Thus, identification of pro-inflammatory markers during ONNV infection on HSF cells may guide therapeutic strategies and pathogenesis investigations. Focusing on a first-line immune response during alphavirus infection, we selected the pro-inflammatory chemokine CCL-2 (or monocyte chemoattractant protein 1, MCP-1) for its active recruitment of monocytes to the site of inflammation, as well as the cytokine IL-1β, which is produced in response to cell infection and tissue injury but also as a marker of arthralgia. Chronic inflammation has been assigned to a prolonged released of cytokines and others proinflammatory mediators, notably CCL-2 and IL-1β, during persistent viral replication in synovial tissue^33^. In the case of ONNV, secretion of pro-inflammatory CCL-2 and IL-1β mediators are involved in the initiation and progression of arthritis^34^. Here, we assess the antiviral and anti-inflammatory effects of phenolics in vitro in human synovial fibroblasts cells (HSF) infected with ONNV including three phenolic acids (gallic acid, GaA; caffeic acid, CaA; chlorogenic acid, ChA), a curcuminoid (curcumin, CU), a flavonoid (quercetin, QU) and a curcuminoid-rich extract (CRE) from turmeric (*Curcuma longa,* Reunion island).

## Materials and methods

### Chemicals

**Figure 1:**
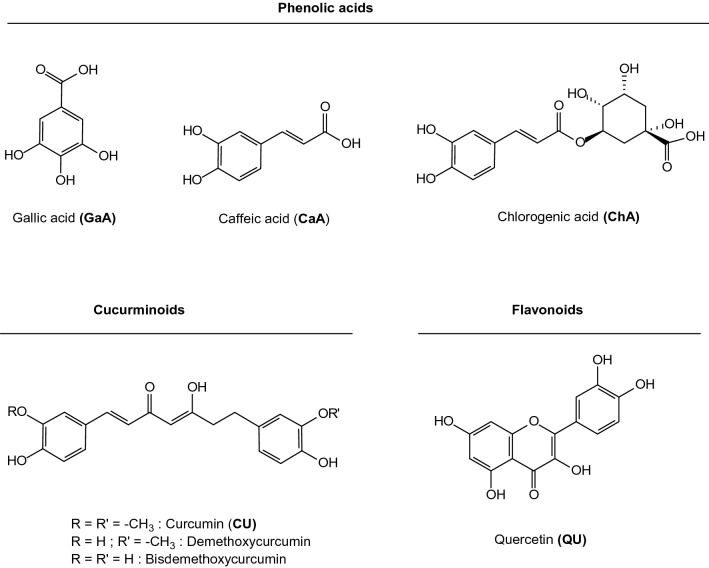
Phenolics with related chemical structural classes.

GaA, CaA, ChA, CU and QU (Fig. 1) were purchased from Sigma-Aldrich and CRE was obtained from turmeric provided by an essential oils company (Extraits de Bourbon, Reunion Island, France). CRE from turmeric was prepared as follows: 2 g of turmeric (*Curcuma longa*) was added to 10 mL of aqueous ethanol (70%, v/v). After incubation at 4 °C for 90 min, sample was centrifuged at 3500 rpm for 20 min at 4 °C. Supernatant was collected and stored at -80 °C until analysis. As previously reported using an ultra-performance liquid chromatography tandem mass spectrometry (UPLC-MS) our analyses led to the identification of curcumin and both derivatives demethoxycurcumin and bisdemethoxycurcumin in CRE sample^35^. The identity and quantity agreed with literature data reported in *Curcuma longa* extracts from India^36^.

### Cell culture and virus

HSF cells were obtained from ScienCell research laboratory (ScienCell, 4700 Clinisciences) and were grown in Modified Eagle's Medium (MEM, PAN Biotech P0408500) supplemented with 10% heat-inactivated fetal bovine serum (FBS; PAN Biotech, 3302 P290907), 2 mM of L-glutamine (Biochrom AG, K0282), 0.1 mg/mL penicillin–streptomycin (PAN Biotech, P0607100), 1 mM of sodium pyruvate (PAN Biotech, P0443100) and 0,5 µg/mL of amphotericin B (PAN Biotech, P0601001). Cells were maintained at 37 °C with 5% CO_2_. A clinical and passage-limited isolate of ONNV was obtained from the National Reference Center (CNR arbovirus, Marseille, France) and titrated on Vero cells at 10^7^ PFU/mL.

To determine the PFU, Vero cells (3 × 10^6^) were plated in plate 6-well. After 24 h incubation, the medium was discarded and 1 mL of diluted viral suspension (dilution factor: 10) was carefully spread over the cell monolayer. 2 h later, 1 mL of carboxyl methyl cellulose (0.4%) in medium was added. Plate was incubated for 2 days at 37 °C with 5% CO_2_. Supernatants were discarded and cells were fixed with Paraformaldehyde (3.7%) 10–20 min. Then, cells were stained with crystal violet (0.5%, Ethanol 20%) to visualize plaque during 5 min. The plates formed were counted and expressed in PFU (Plate Forming Unit)/mL.

### Cell infection and/or treatment

HSF cells were placed in a 96-, 24- or 6-well plates and maintained at 37 °C in a humid atmosphere with 5% CO_2_. The medium was replaced twice a week. Cells were allowed to grow to 80–90% confluency. Cells were then infected by ONNV to multiplicity of infection 1 (MOI 1) in the presence or not of phenolics (10 µM) for 24 h at 37 °C in a humid atmosphere with 5% CO_2_. In order to infect at MOI 1, cells were counted before each infection. HSF cells were counted with a hemocytometer using trypan blue exclusion. method^37^.

### Cytotoxicity assay

Cytotoxicity assay were performed by quantitative release of lactate dehydrogenase (LDH) from damaged cells using a colorimetric-based kit (ref. G1781, CytoTox 96 Non-Radioactive Cytotoxicity Assay, Promega). Cells were grown as previously described and infected with escalating MOIs of ONNV (MOI 10^–3^ to 1) or treated with phenolics (1 to 100 µM) during 24 h. Culture medium was collected and cells were lysed following the manufacturer's instructions. Released LDH in culture medium after treatments was compared to the maximum LDH release (intracellular LDH induced by addition of Triton 1%). Cytotoxicity was expressed relative to maximum LDH release with the formula: % cytotoxicity = 100 × experimental LDH release/maximum LDH release Control (CTRL; refers to LDH release from untreated cells).

### Immunofluorescence staining and microscopy

Cells were grown on glass coverslips in a 24-well plate as previously described, then treated with phenolics (10 µM) and exposed or not to ONNV (MOI 1) for 24 h. Coverslips were then fixed and permeabilized with cold 99% ethanol at room temperature for 10 min. Cells were incubated overnight with primary antibody Alpha SC 293,153 anti-alphavirus capsid (3582, Santa Cruz Biotechnology). Alexa Fluor488-conjugated donkey anti-mouse was used as secondary antibody (A-21202, Invitrogen, Thermo Fischer Scientific). Nuclei were revealed by staining with the nuclear fluorochrome 4′,6-diamidino-2-phenylindole (DAPI, Sigma-Aldrich, Germany). The coverslips were mounted with Vectashield (Vector Labs; Clinisciences) and fluorescence was observed using a Nikon Eclipse E2000-U microscope (Nikon, Tokyo, Japan). Images were captured and processed using a Hamamatsu ORCA-ER camera and the imaging software NIS-Element AR (Nikon, Tokyo, Japan). Magnification X20.

### ELISA assay

Culture media collected from cells infected (MOI 1) co-treated or not with phenolics (10 µM) as previously described, was analyzed by ELISA. IL-1β and CCL-2 concentrations in HSF cells supernatants were measured using commercially available ELISA kits for CCL-2 (Peprotech, cat. no. 900-T31) and IL-1β (Peprotech, cat. no. 900-K95), according to the manufacturer’s instructions. Samples were analyzed from three independent biological experiments.

### qRT-PCR analysis

Total RNA from HSF cells exposed to phenolics (10 µM) in the presence or absence of ONNV (MOI 1) during 24 h, was extracted directly from harvested cell culture (in 6-well plates) using a QIAamp RNA Blood Mini Kit (QIAGEN, Cat No 52304). 350 μL of lysis buffer from the kit was added to each well (culture media were collected before), collected after 5 min and kept at – 80 °C until use. qRT-PCR experiments were done using the One Step Prime Script Syber Green RT-PCR kit from TAKARA (Cat No RR066A). qRT-PCR was performed in a final volume of 5 μL containing 1 μL of extracted total RNA per reaction, 2.7 μL of enzyme mix and 1.3 μL of primers mix with final primer concentration of 250 nM. qRT-PCR was carried out in Quantstudio 3 PCR thermocycler (Thermo Fisher Scientific) with the following steps: a reverse transcription at 42 °C for 5 min and 40 cycles comprising a denaturation step at 95 °C for 5 s, annealing step at 58 °C for 15 s and extension step at 72 °C for 15 s. Fluorescence data were collected at 520 nm during the extension step. Relative gene expression was calculated using GAPDH as a reference gene^38^. Samples were analyzed from three independent biological experiments. Primer sequences related to the genes coding for CCL-2, IL-1β, E1, E2, nsP1, nsP2 and GAPDH are listed in Table 1 below.

**Table 1:** Primers used for qRT-QPCR analysis.

Target gene	Forward	Reverse	Detection
CCL-2	CTGCTCATAGCAGCCACCTT	CTTGAAGATCACAGCTTCTTTGGG	Sybergreen
IL-1β	TTGCTCAAGTGTCTGAAGCAG	GGTGGTCGGAGATTCGTAGC	Sybergreen
E1	CACCGTCCCCGTACGTAAAA	GGCTCTGTAGGCTGATGCAA	Sybergreen
E2	CCCCTGACTACACGCTGATG	CCTTCATTGGAGCCGTCACA	Sybergreen
nsP1	GAGAAAACTTGCGTCAGCCG	GACGGCGTAGACGTCTTGAT	Sybergreen
nsP2	GCGGAGCAGGTAAAAACGTG	TAGAACACGCCCGTCGTATG	Sybergreen
GAPDH	TGCGTCGCCAGCCGAG	AGTTAAAAGCAGCCCTGGTGA	Sybergreen

### Statistical analysis

Data were expressed as means ± SEM. All assays were performed in triplicate independent biological experiments. Statistical analysis was achieved using Graph Prism 3 software. Significant differences (*p* < 0.05) between the means were determined by analysis of variance (two-way ANOVA) procedures followed by a multiple comparison test (Bonnferroni).

## Results

### Cytotoxicity assessment

**Figure 2:**
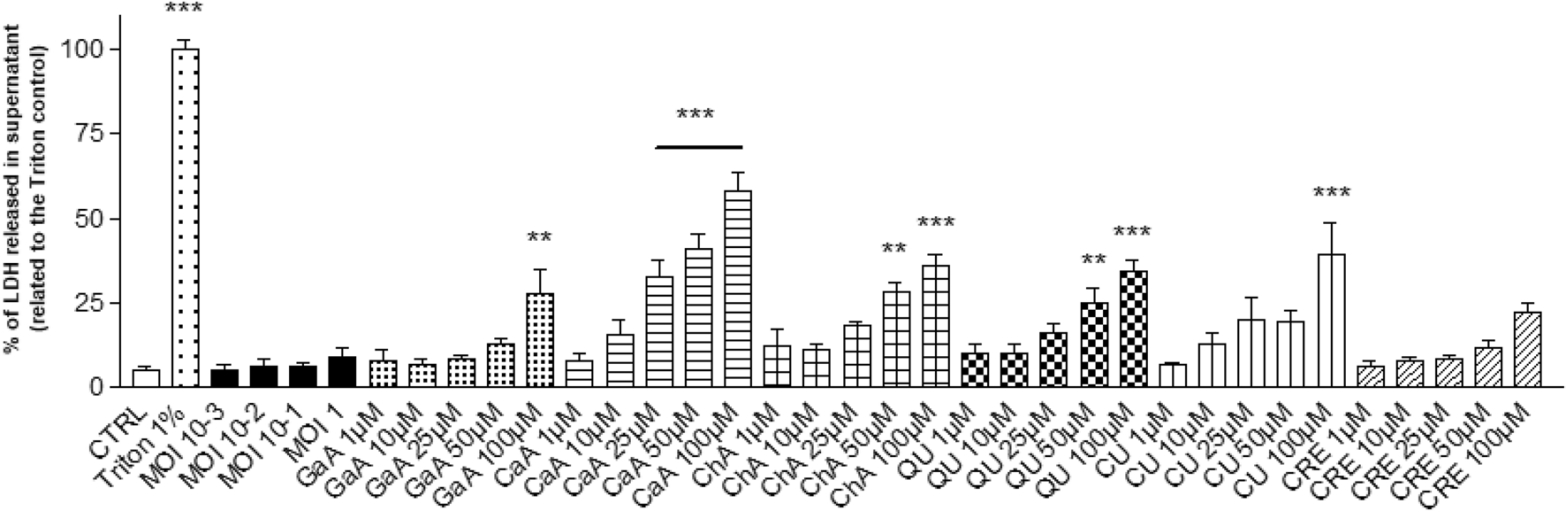
Concentration-dependent cytotoxic effects of phenolics or ONNV on cultured HSF cells. Cells were infected by ONNV (MOI 10^–3^ to 1) or phenolics (1 to 100 µM) for 24 h. Released LDH in culture medium was measured using a colorimetric-based method (CytoTox 96 Non-Radioactive Cytotoxicity Assay) and expressed relative to the maximum release by application of a lysis buffer (Triton 1%). Reported values are means ± SEM of three independent experiments and p value was calculated using the Bonferroni multiple comparison test (**p < 0.01; ***p < 0.001) with untreated cells (CTRL).

LDH release assays was performed to determine the capacity of ONNV and phenolic to affect HSF cells viability after 24 h of exposure. As shown in Fig. 2, a basal release of LDH (5.10 ± 1.28) was found in culture supernatants of untreated cells (CTRL). Escalating MOI (10^–3^ to 1) of ONNV did not significantly affect LDH release from HSF cells when compared to the basal release (CTRL). In contrast, a dose-dependent effect was observed by increasing phenolics concentrations (1 to 100 µM). Significant LDH release compared to untreated cells (CTRL) was observed for the highest doses of gallic acid (100 µM; *p* < 0.01), caffeic acid (> 25 µM; *p* < 0.001), chlorogenic acid (> 50 µM; *p* < 0.01 and 0.001), quercetin (> 50 µM; *p* < 0.01 and 0.001) and curcumin (100 µM; *p* < 0.01), while CRE led to an insignificant effect on LDH release for all the tested concentrations. Based on the overall results, the further experiments on HSF cells were performed using ONNV at a MOI of 1 (to allow the virus to infect all cells) for infection by ONNV and the concentration of 10 µM (the highest concentration that can be found in the bloodstream that does not show a cytotoxic effect in our results) for treatment with phenolics^39^.

### Reduction of ONNV infection

**Figure 3:**
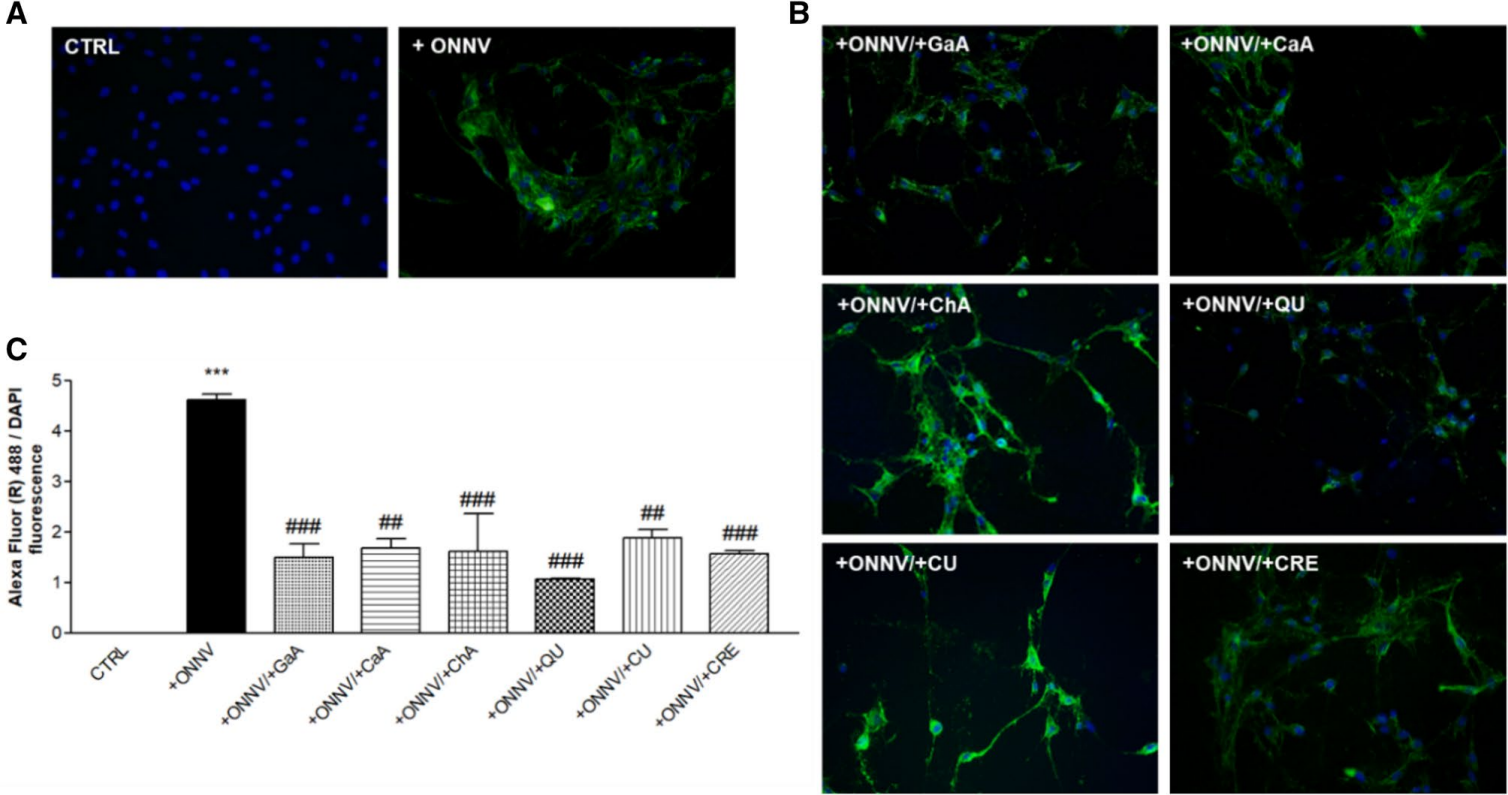
Quercetin impairs HSF cells infection by ONNV. HSF cells were exposed to ONNV (MOI 1) co-treated or not with phenolics (10 µM) for 24 h. Cells were probed with primary antibody Alpha SC 58,088 anti-alphavirus before nuclei staining by DAPI (blue) and antibody binding by Alexa Fluor 488-conjugated donkey anti-mouse IgG antibody (green) (**A**). Untreated cells (CTRL) and infected cells (+ ONNV) (**B**) Alexa Fluor 488/DAPI overlay upon phenolic treatment. (**C**) Ratio Alexa Fluor 488 / DAPI fluorescence intensity with at least 10,000 DAPI stained cells. Reported values are means ± SEM of three independent experiments. *p* value was calculated using the Bonferroni multiple comparison test (***p < 0.001) between infected cells (+ ONNV) and untreated cells (CTRL) or (^##^*p* < 0.01, ^###^*p* < 0.001) between infected cells (+ ONNV) and infected cells upon treatment with phenolics.

Immunofluorescence and microscopy were used to study the effect of the phenolics on HSF cells infection by ONNV. Overlay micrographs reported in Fig. 3 were obtained from DAPI staining of nuclei (blue) and fluorescent specific alphavirus capsid antibody binding (green) (Fig. 3A). As shown in Fig. 3B, ONNV infected cells and treated by phenolics visually led to a reduced viral antigen detection, as particularly evidenced for quercetin (+ ONNV/ + QU). Ratio Alexa Fluor 488 / DAPI fluorescence intensity (Fig. 3C) confirmed the micrograph data given that infected cells had an overall staining level of (+ ONNV: 4.61 ± 0.09, *** *p* < 0.001) when compared infected-QU-treated cells (+ ONNV/ + QU: 1.06 ± 0.02, ### *p* < 0.001). Moreover, we found that all other phenolics significantly decreased viral antigen detection in HSF cells. (### *p* < 0.001 for GaA, ChA, CRE; ## *p* < 0.01 for CaA and CU).

### Reduction of viral RNA levels

**Figure 4:**
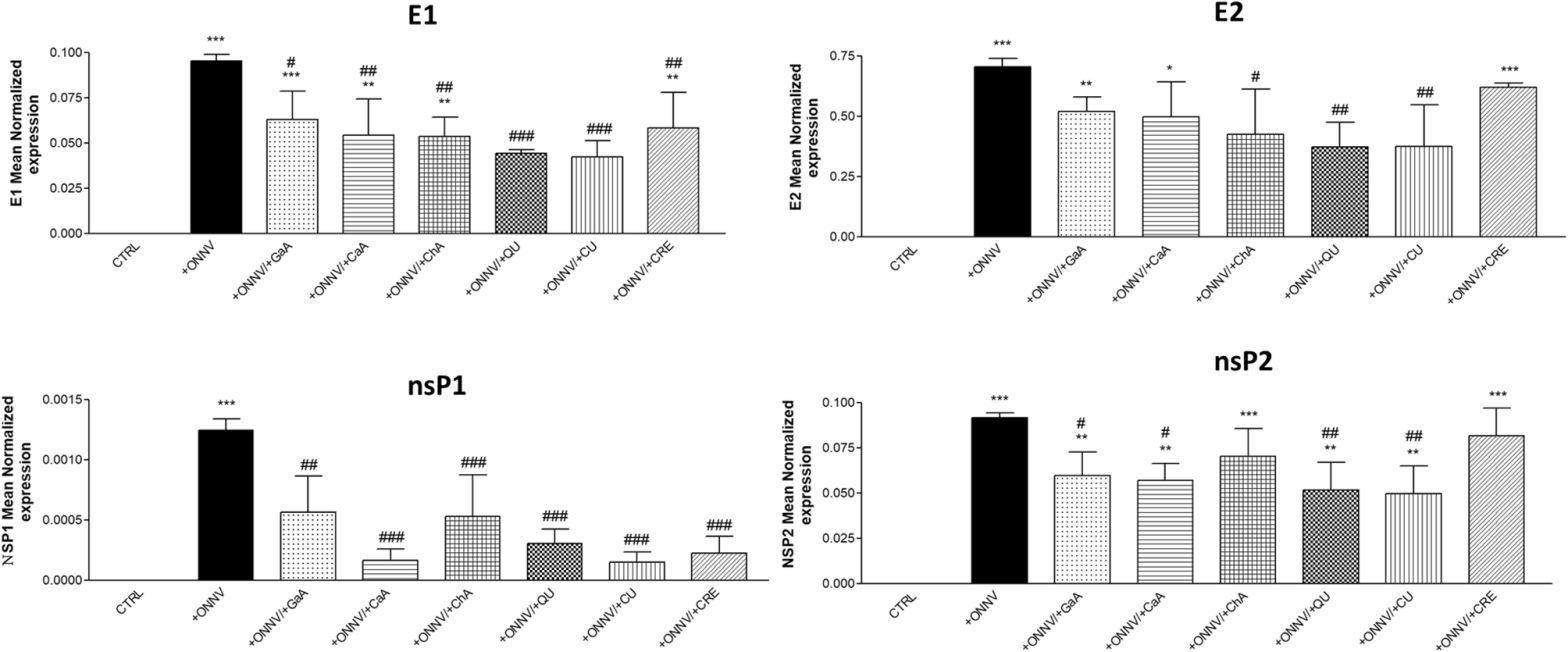
Quercetin impairs ONNV replication in HSF cells. HSF cells were infected by ONNV (MOI 1) co-treated or not with phenolic compounds (10 µM) for 24 h. Then, RNA was collected and E1, E2, nsP1 and nsP2 gene expression levels were determined by qRT-PCR. Reported values are means ± SEM of three independent experiments. *p* value was calculated using the Bonferroni multiple comparison test: *p < 0.05, **p < 0.01, ***p < *0.001* when compared to non-infected and non-treated cells (CTRL) and ^#^*p* < 0.05, ^##^*p* < 0.01, ^###^*p* < 0.001 when compared to infected cells (+ ONNV).

We assessed by qRT-PCR the effect of phenolics (10 µM) on viral RNA levels (E1, E2, nsP1 and nsP2) in ONNV-infected HSF cells (MOI 1) as well as in the supernatants. First in cells and as shown in Fig. 4, viral RNA levels increased significantly in infected cells (+ ONNV) for all tested genes (***p < 0.001). Treatment with phenolics of uninfected cells was carried out to exclude possible cross-contamination during experiments. No viral RNA was identified in these samples (data not shown). In infected cells, the phenolics were able to reduce viral RNA significantly as compared to the positive control (+ ONNV alone). E1 and nsP1 gene expression levels were significantly decreased by all tested phenolics. No significant reduction of viral RNA levels was observed when applied GaA, CaA or CRE for E2, and ChA or CRE for nsP2. A cross-analysis of these results indicates a broad capacity of QU and CU to reduce all viral RNA levels, and more intensively nsP1 (QU: 0.001 ± 9.20*10–5 to 0.0003 ± 0.0001, ### *p* < 0.001; CU: 0.001 ± 9.20*10–5 to 0,0001, ### *p* < 0.001).

**Figure 5:**
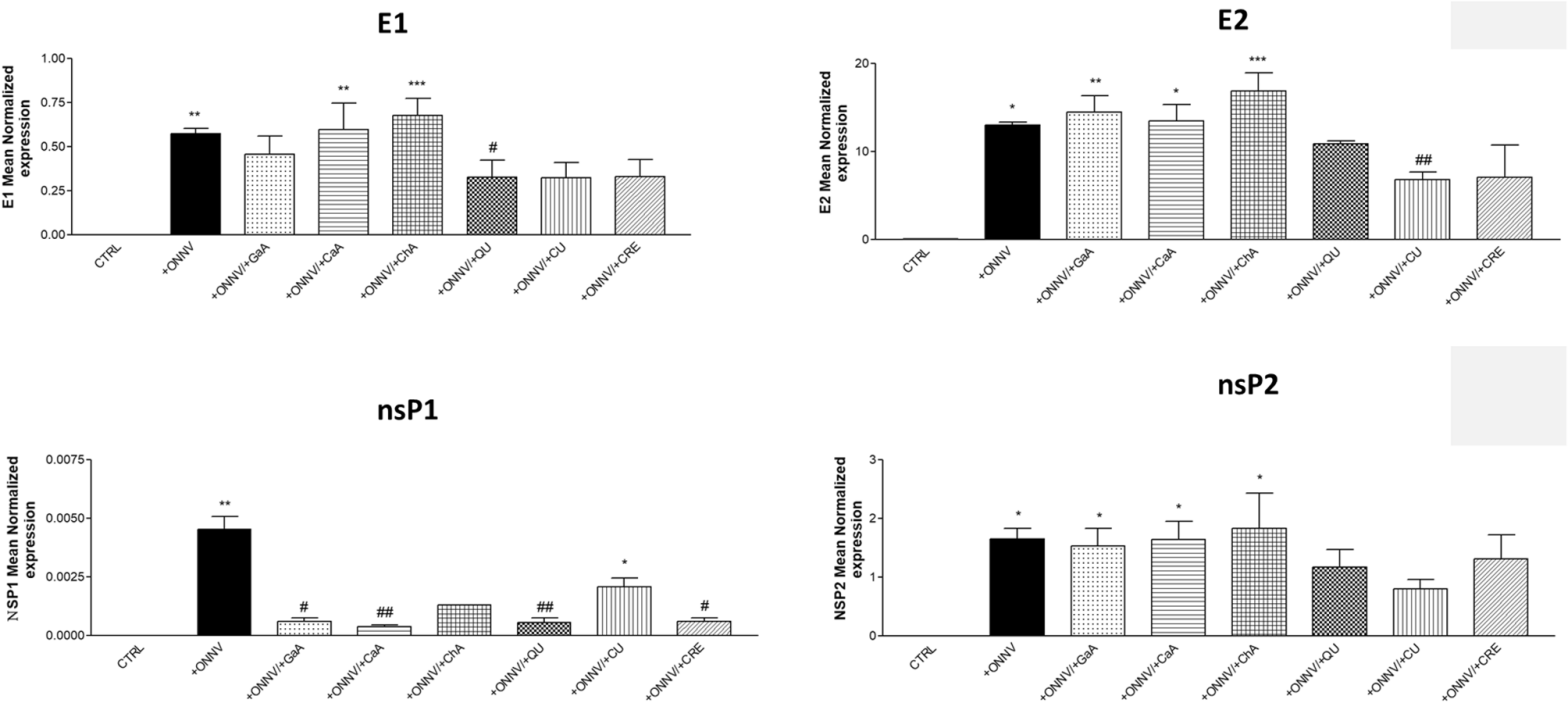
Quercetin impairs ONNV replication in supernatants in HSF cells. HSF cells were infected by ONNV (MOI 1) co-treated or not with phenolic compounds (10 µM) for 24 h. Then, RNA was collected in the supernatants and E1, E2, nsP1 and nsP2 gene expression levels were determined by qRT-PCR. Reported values are means ± SEM of three independent experiments. p value was calculated using the Bonferroni multiple comparison test: *p < 0.05, **p < 0.01, ***p < *0.001* when compared to non-infected and non-treated cells (CTRL) and ^#^*p* < 0.05, ^##^*p* < 0.01 when compared to infected cells (+ ONNV).

For the experiments testing viral RNA levels in supernatants ONNV infection and replication was evidenced for all tested genes Fig. 5 (*p < 0.05 and **p < 0.01). Only QU was able to counteract ONNV infection by decreasing Viral RNA for both E1 (0.0045 ± 0.0005 to 0.0005 ± 0.0002, ## *p* < 0.01) and nsP1 (0.57 ± 0.03 to 0.32 ± 0.09, # *p* < 0.05). No statiscal significant effect were observed for E2 and nsP2.

### Downregulation of CCL-2 and IL-1β pro-inflammatory mediators

**Figure 6:**
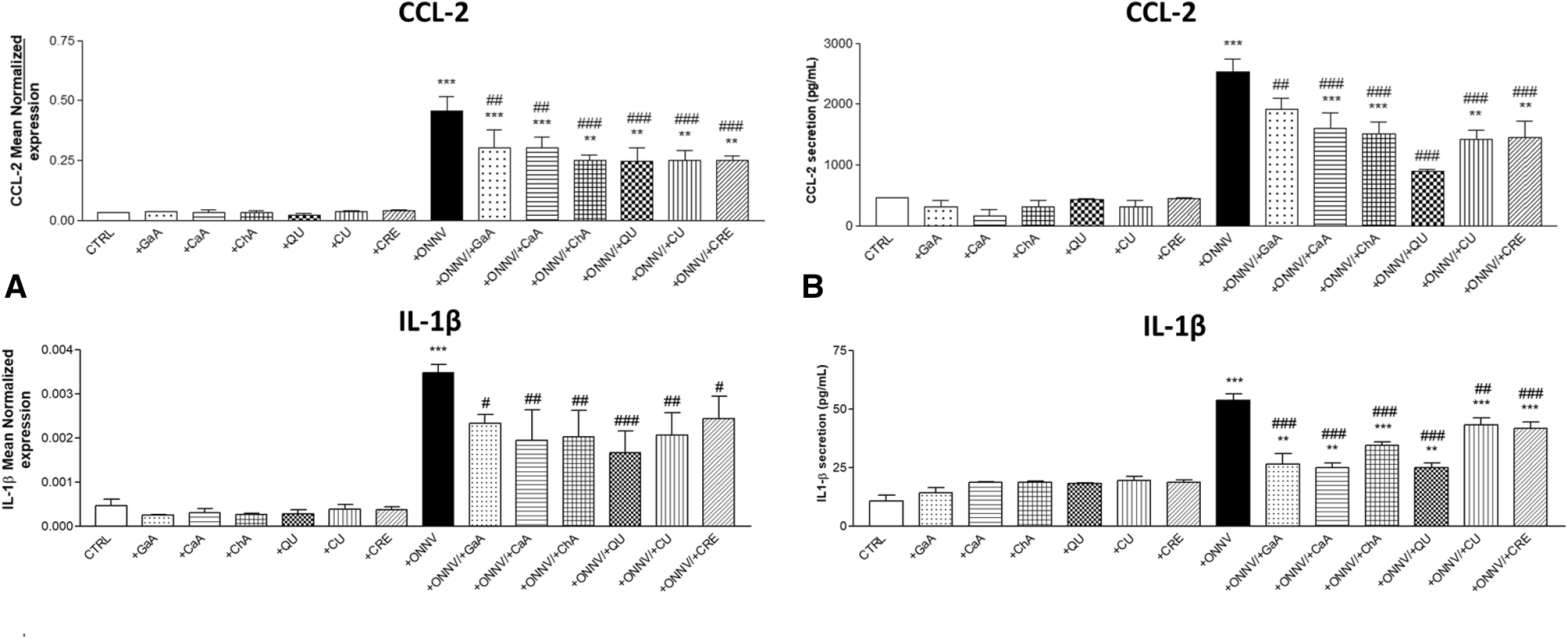
Quercetin reduces CCL-2 and IL-1β pro-inflammatory mediators production in ONNV infected HSF cells. HSF cells were infected by ONNV (MOI 1) and/or treated with phenolics (10 µM) for 24 h. Then, RNA was collected and CCL-2 and IL-1β gene expression levels were determined by qRT-PCR, (**A**) Pro-inflammatory mediators RNA levels. Then, culture media were collected CCL-2 and IL-1β levels evaluated by ELISA, (**B**) Pro-inflammatory mediators levels. Reported values are means ± SEM of three independent experiments. p value was calculated using the Bonferroni multiple comparison test: *p < 0.05, **p < 0.01, ***p < *0.001* when compared to non-infected and non-treated cells (CTRL) and ^*#*^*p* < *0.05, *^*##*^*p* < *0.01, *^*###*^*p* < *0.001* when compared to infected cells (+ ONNV).

ONNV causes an arthralgia-rash and inflammatory syndrome that can persist for months. The inflammation is produced by a release of pro-inflammatory chemokines and cytokines such as IL-1β and CCL-2 which are involved in the initiation and progression of arthritis^34^. qRT-PCR and ELISA assays were performed to determine levels of pro-inflammatory chemokine CCL-2 and cytokine IL-1β when phenolics (10 µM) were applied on non-infected or infected cells (MOI 1). As shown in Fig. 6A,B, significantly increased levels of both gene expression and secretion of CCL-2 and IL-1β were observed in infected cells (+ ONNV) when compared to the basal level from non-treated and non-infected cells (CTRL). Phenolics are not affecting the inflammatory status on their own. Indeed, they caused no significant change in CCL-2 and IL-1β expression and secretion levels in non-infected cells (+ GaA, + CaA, + ChA, + QU, + CU or + CRE) in comparison to control (CTRL). Application of phenolics in infected cells resulted in significant downregulation of both gene expression and secretion levels in all cases when compared to infected cells (+ ONNV). Analyses of these data demonstrated a broad ability of QU to reduce viral RNA and to exert anti-inflammatory activity by reducing the expression and secretion of two cytokines tested (*p* < 0.001).

## Discussion

The lack of specific treatment for ONNV infection makes the identification of readily available, safe and efficient antiviral agents a great imperative. Phenolics are of growing interest for their broad-spectrum antiviral activity^40^ and for their anti-inflammatory potentiality in chronic diseases^41^. The aim of this study was to evaluate the effects of phenolics against ONNV infection in HSF cells. Our major result ascertained the peculiar capacity of the flavonol (QU) to control for viral RNA level and the inflammation following exposure to ONNV.

Synovial fibroblasts are the main target for alphaviruses infections including during the chronic stage with persistent inflammation and arthritic disease. This makes synovial fibroblasts an appropriate cell model to assess both acute and chronic alphavirus infection and treatment, as demonstrated previously in our team^21^. Our present in vitro experiments showed that viable HSF cells exposed to ONNV infection up to MOI 1 (Fig. 2) are appropriate for study of viral replication (Figs. 3, 4 and 5) and major pro-inflammatory reactions by CCL-2 and IL-1β markers (Fig. 6). Thus, HSF cells can be used to achieve efficient assessment of phenolics (10 µM) on critical endpoints for an antiviral and/or anti-inflammatory strategy against ONNV.

Phenolics have been established as ‘functional nutrients’ for their health benefits^42^ and QU is the most abundant phenolic provided by human diet^43^. Our overall results on HSF cells support the reduction of viral RNA and anti-inflammatory capacities of phenolics at 10 µM against ONNV, without significant toxicity. However, the benefits of phenolics in medicinal use rely on their bioavailability that refers to their plasmatic concentrations after food ingestion, mostly ranging from 0.072 to 5 μM^44^. The bioavailability of quercetin is considered as significant but strongly affected by many factors^45^, thereby requiring high dosage forms in a pharmaceutical development to obtain the relevant plasmatic concentrations for expected biological effects. An improved formulation of quercetin for oral absorption has been recently described from a clinical study^46^. Additionally, quercetin at concentration up to 10 µM inhibits the ATPase of proteins associated with multi-drug resistance^47^. This may support the applicability of quercetin for pharmaceutical purposes.

**Figure 7:**
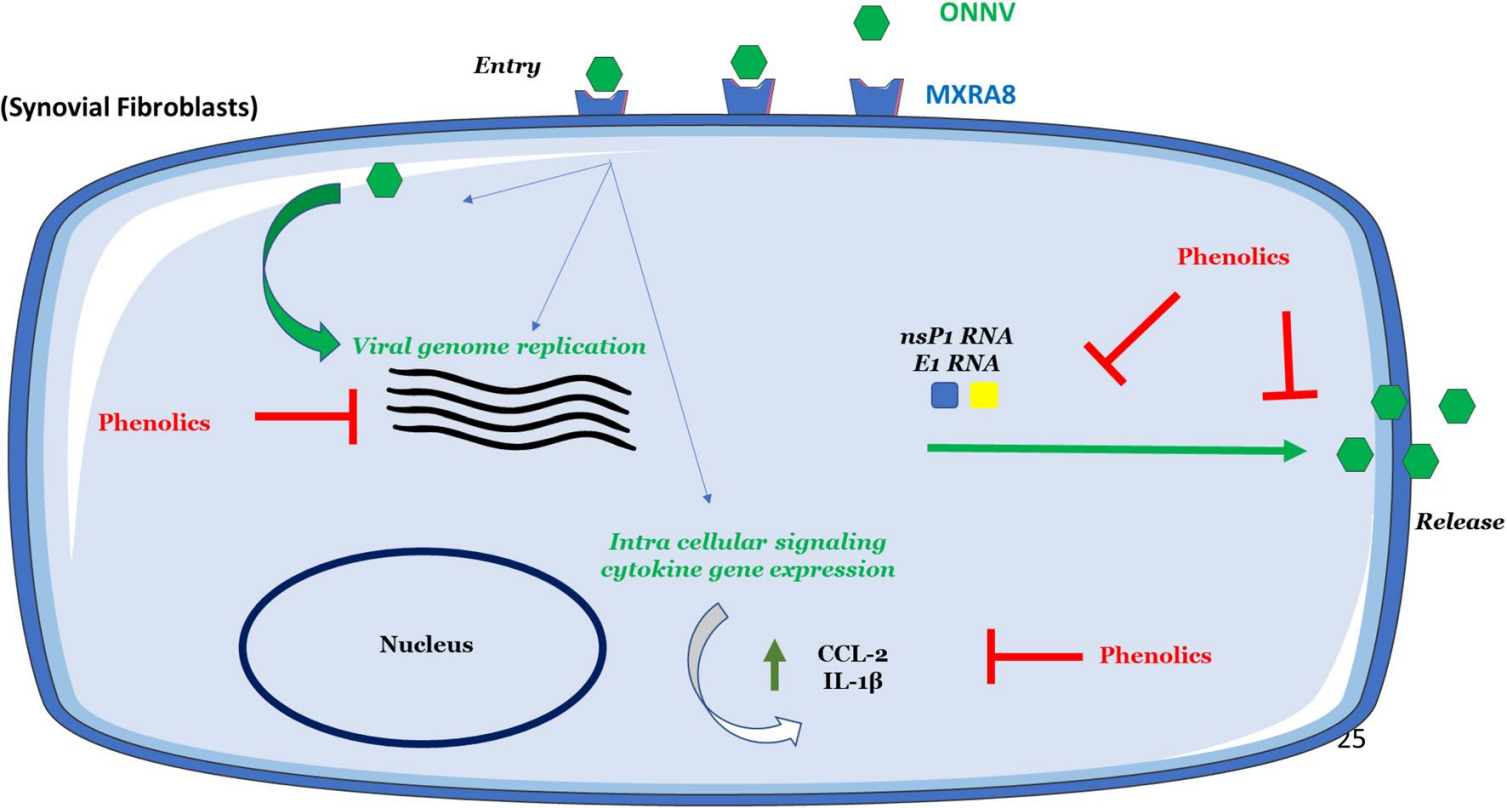
Antiviral mechanism of Phenolics.

Broad-spectrum antiviral activity of quercetin has been reported for hepatitis C virus^48^, porcine epidemic diarrhea virus^49,50^, herpes simplex virus type 1 and type 2^51^, rhinovirus^52^ and dengue virus type 2^53^. Quercetin remains attractive against (re)-emerging pathogens including Mayaro virus^54^, influenza A virus^55^, CHIKV^56^, Middle East respiratory syndrome coronavirus^57^, the severe acute respiratory syndrome coronavirus^58^ and it’s more recent worldwide epidemic form SARS-CoV-2^59^. To the best of our knowledge, quercetin antiviral capacity has not been reported yet against ONNV infection. Our results show that quercetin at 10 µM may impair viral infection in HSF cells. The broad-range activities of quercetin support its capacity to interfere with major key-points of the replication cycle but few data are available in the case of alphaviruses^60^. Inhibition of the viral replication was previously demonstrated for Mayaro virus in Vero cells^54^. In contrast, increasing doses (6.25–200 µg/mL) of quercetin were proved to be crudely less efficient than the phenolic silymarin to inhibit CHIKV-induced cytopathic effects (CPE) in Vero and BHK-21 (Baby hamster kidney) cells^56^. As first evidence from our results, down-regulation of viral replication occurs for ONNV in HSF cells using quercetin which, therefore, may operate as inhibitor of the viral replication. Alphaviruses are small enveloped RNA viruses whose genome consists of a single-stranded, positive-sense mRNA. The non-structural polyprotein of this genome is cleaved into four different proteins (nsP1, nsP2, nsP3 and nsP4) that are necessary for the transcription and translation of viral mRNA in the cytoplasm of host cells. The structure of the nsP1 protein, which is an mRNA-capping enzyme, is essential for the translation of viral mRNA^61^. nsP1 is involved in the recruitment of other nsps^62^ and its membrane association with the host cell is crucial for the replication of the virus^63^. Therefore, nsP1 is an interesting target for drug development. Additionally, the marked effect of quercetin on nsP1 is of interest for its druggable status claimed for CHIKV^64,65^. Our data suggest that phenolics and especially QU act on nsP1. They could act either by modifying the structure of nsP1 which would disrupt the translation of viral RNA, or by disrupting the affinity of nsP1 for the cell membrane which would stop the recruitment of other nsPs, thus preventing viral replication (Fig. 7).

The identification of pro-inflammatory probes during ONNV infection on HSF cells may guide therapeutic strategies and pathogenesis investigations. Focusing on a first-line immune response during alphavirus infection, we selected the pro-inflammatory chemokine CCL-2 (or monocyte chemoattractant protein 1, MCP-1) for its active recruitment of monocytes to the site of inflammation, as well as the cytokine IL-1β produced in response to cell infection and tissue injury but also a marker of arthralgia. Chronic inflammation has been assigned to a prolonged released of cytokines and others proinflammatory mediators, noticeably CCL-2 and IL-1β, during persistent viral replication in synovial tissue^33^. In the case of ONNV, secretion of pro-inflammatory CCL-2 and IL-1β mediators are involved in the initiation and progression of arthritis^34^. We showed that ONNV infection of HSF cells increases the levels of both CCL-2 and IL-1β, but treatment with phenolics results in their downregulation (Fig. 6). The marked effect of quercetin was consistent with its reported prominent capacity to alleviate chronic inflammation by different ways^66^.

## Conclusion

Our unprecedent findings are related to the capacity of common phenolic compounds to reduce viral replication of ONNV and inflammation occurring on the key effectors HSF cells. We gave evidence for the high value of the flavonol quercetin to achieve at low and non-toxic dose both inhibition of viral replication and downregulation of two key pro-inflammatory markers (CCL-2 and IL-1 β). This study provides preliminary assessment of the role of phenolic treatment in alleviating ONNV infection in vitro. Further studies are required to confirm the use of phenolics in treating ONNV infection both in vitro and in vivo*,* as well as investigations to assess the potential action of quercetin (QU) against other viruses.

## Abbreviations

CaA: Caffeic acid
ChA: Chlorogenic acid
CRE: Curcuminoids-rich extract
CU: Curcumin
CHIKV: Chinkungunya virus
GaA: Gallic acid
DAPI: 4′,6-Diamidino-2-phenylindole
HSF: Human synovial fibroblasts
LDH: Lactate dehydrogenase
MOI: Multiplicity of infection
ONNV: O’nyong-nyong virus
PFU: Plaque Forming Unit
QU: Quercetin
RT-PCR: Real-time polymerase chain reaction
qRT-PCR: Quantitative RT-PCR
ELISA: Enzyme-linked immunosorbent assay
